# Supplementary material for: A Global Screen for Assembly State Changes of the Mitotic Proteome by SEC-SWATH-MS
Source: Cell Syst. 2020 Feb 26;10(2):133–155.e6. doi: 10.1016/j.cels.2020.01.001 (PMC7042714; doi:10.1016/j.cels.2020.01.001)

P68036 | UB2L3_HUMAN | UBE2L3 UBCE7 UBCH7
Monomer MW [kDa]: 17.862 Monomer expected elution fraction: 55

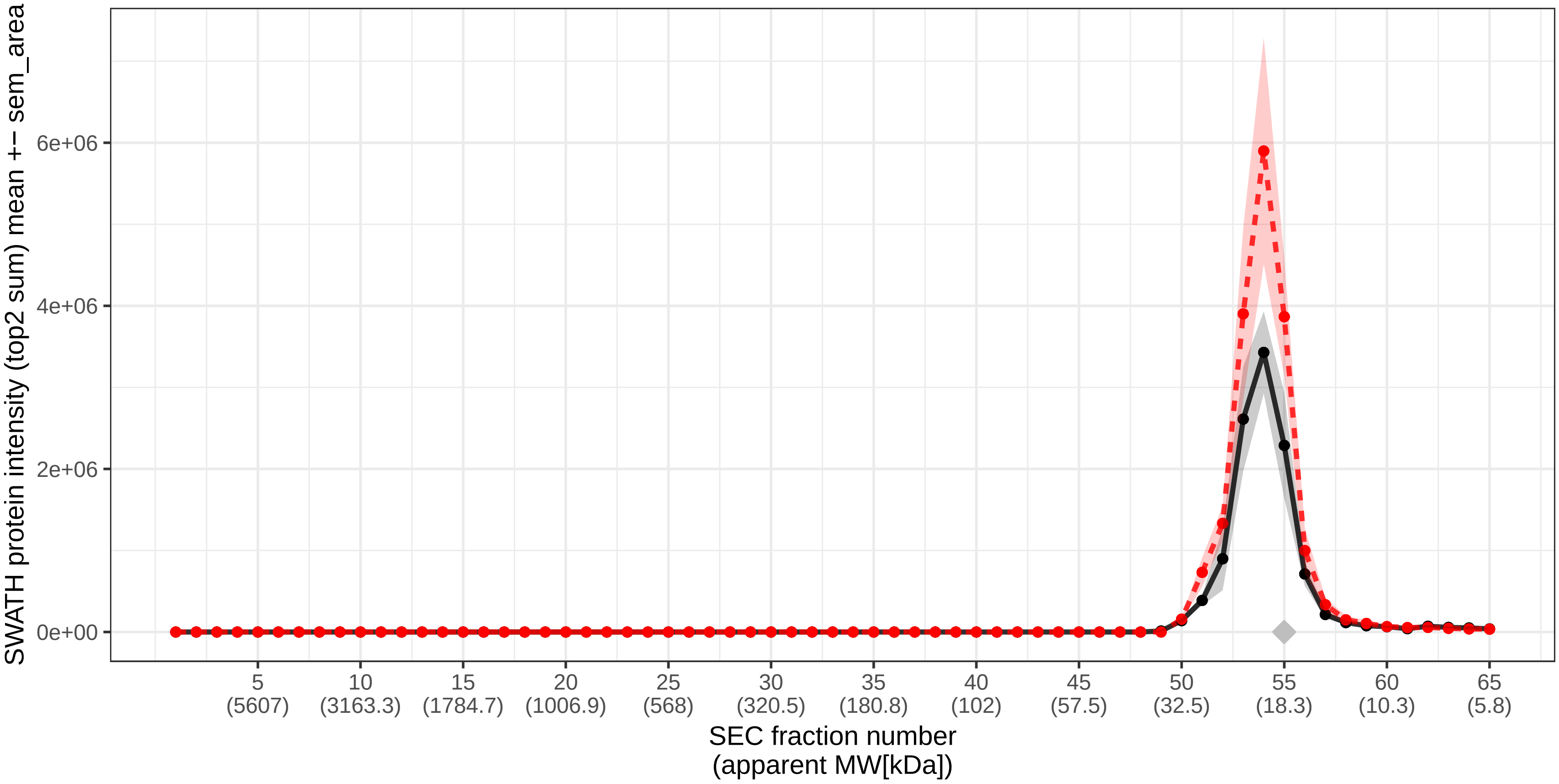

Supplement: Data S1. SEC-SWATH-MS Protein Chromatograms, Related to Figure 1 [file mmc6.zip › SECchrom_P68036_UB2L3_HUMAN_UBE2L3_UBCE7_UBCH7.pdf]
